# Intercomparison study on commonly used methods to determine microplastics in wastewater and sludge samples

**DOI:** 10.1007/s11356-019-04584-6

**Published:** 2019-03-02

**Authors:** Mirka Lares, Mohamed Chaker Ncibi, Markus Sillanpää, Mika Sillanpää

**Affiliations:** 1 Department of Green Chemistry, School of Engineering Science, Lappeenranta-Lahti University of Technology LUT, Sammonkatu 12, FI-50130 Mikkeli, Finland; 2 Ecotoxicology and Risk Assessment, Laboratory Centre, Finnish Environment Institute, Ultramariinikuja 4, FI-00430 Helsinki, Finland

**Keywords:** Microplastics, WWTP, Wastewater, Sludge, Recovery, Identification

## Abstract

The harmonized procedures in terms of the sampling, sample treatment and identification of microplastics in different environmental samples are missing, which poses challenges to researchers to compare the results or to adopt ‘the most effective’ monitoring approach. Furthermore, in the related literature, the used procedures are rarely tested with spiked microplastics to predetermine their recovery rates. Without this knowledge, results should only be discussed as rough estimations of the real environmental concentrations of microplastics. In this study, six different methods previously used in microplastic studies of different media were tested with municipal wastewater and digested sludge samples, spiked with seven different types of plastic particles and fibres. Recovery rates, time consumption, advantages and disadvantages were assessed and most suitable treatment procedures (i.e. high recovery rates in short amount of time) were chosen for both wastewater and sludge. Suitability of staining with Rose Bengal was examined together with most efficient methods, but it did not improve the recovery of microplastics. In addition, the possible impacts of the treatments for identification with micro-Raman and FTIR microscope were assessed. Filtration with size fractioning was found to be the best method for both wastewater and sludge samples, with recovery rates of spiked microplastics around 91.4% and 92.9%, respectively.

**Electronic supplementary material:**

The online version of this article (10.1007/s11356-019-04584-6) contains supplementary material, which is available to authorized users.

## Introduction

Since plastic production and consumption is accelerating (PlasticsEurope 2016), more plastics are also discharged to both terrestrial and aquatic environment. Plastics may become shredded into smaller fragments during the use or after reaching the environment as a result of different kind of weathering and physical breakdowns (Arthur et al. 2009). As well, they can be purposely produced as small particles, for example for plastic industry or cosmetic use (Fendall and Sewell 2009). Until now, these microplastics (MPs) have been examined in various environments, and related studies confirmed their presence in seawater (Andrady 2011; Magnusson and Norén 2014), freshwater (Fischer et al. 2016), sediments (Browne et al. 2011; Hidalgo-Ruz et al. 2012), fishes (Lusher et al. 2017), arctic sea ice (Peeken et al. 2018) and even in the air (Dris et al. 2016).

As the research effort on MPs monitoring has been accelerating during recent decade, a great variety of sampling and treatment methods have been introduced (Hidalgo-Ruz et al. 2012; Horton et al. 2017; Lusher et al. 2017). Nonetheless, despite the great number of MPs-related studies, the efficiencies of the used method and effects of different thermal, chemical or physical treatments on MPs samples are rarely assessed and reported together with the MPs concentrations (Underwood et al. 2017). As for any pollutant investigations, without knowledge of the actual recovery rates, there is a risk of under- or overestimation of the reported MPs concentrations.

Even though many researchers are emphasizing the need for standardized and optimized protocols for environmental sampling (Horton et al. 2017; Lusher et al. 2017; Underwood et al. 2017), the variability of sample matrices (e.g. wastewater, sludge, sediment, biota) necessitates the establishment of specified methods for different types of samples (Hale 2017). Furthermore, sampling, preparation and detection should be optimized based on the aim of the study (Hale 2017). For example, if additives or contaminants attached to MPs are supposed to be studied, samples should not be treated with highly alkaline or acidic chemicals (e.g. H_2_SO_4_ or KOH), which might destroy or remove the substances of interest (Rios Mendoza et al. 2016).

Because MPs are formed from a various group of polymers and differently shaped particles, environmental MPs may have a high variation in their compositions, which should be taken into account with selected treatment methods. Some polymers are known to be more sensitive to acidic solutions or higher temperatures than others (Lusher et al. 2017). Different polymers have also different densities, varying from 0.9 g/cm^3^ for polypropylene (PP) to 1.6 g/cm^3^ for polyvinylchloride (PVC) and polyoxymethylene (POM) (Hidalgo-Ruz et al. 2012), which might have an effect on the results from density-based separation methods. In addition, various research investigations concurred that microplastic fibres (MPFs) are the most abundant types of MPs in the marine environment (Browne et al. 2011; Magnusson and Norén 2014; Setälä et al. 2016; Barrows et al. 2017; Leslie et al. 2017; Talvitie et al. 2017a). Nevertheless, fibres have been excluded from most of the studies concerning recovery rates of MPs. Therefore, most of the reported recovery rates from the related literature are only relevant to MP particles (spheres, fragments, etc.), and thus systematically underestimating the pollution threat caused by MPs (Nuelle et al. 2014; Masura et al. 2015; Dyachenko et al. 2017; Karami et al. 2017; Mahon et al. 2017; Quinn et al. 2017; Ziajahromi et al. 2017; Lares et al. 2018).

To the best of our knowledge, the most inclusive testing for recovery rates and suitability of different chemicals in MP analyses was conducted by Karami et al. (2017). They studied suitable procedures for examining MPs from fish samples and included low-density polyethylene (LDPE), high-density polyethylene (HDPE), polypropylene (PP), polystyrene (PS), polyethylene terephthalate (PET), polyvinylchloride (PVC), nylon-6 (PA 6) and nylon-66 (PA 66) to their experiments with sizes mostly under 300 μm. Generally, most of the researchers have still relied on one or two polymers as representatives of all microplastics in recovery rate testing (Masura et al. 2015; Dyachenko et al. 2017; Ziajahromi et al. 2017; Lares et al. 2018).

In addition, effluents of wastewater treatment plants (WWTPs) were proven to be a route for MPs to reach the environment, as they collect MP-containing discharges from both industrial and municipal wastewaters (Talvitie et al. 2015; Murphy et al. 2016; Talvitie et al. 2017a; Lares et al. 2018). In WWTPs, most of the MPs have been reported to be concentrated into sludge (Murphy et al. 2016; Talvitie et al. 2017b; Lares et al. 2018), which is often used as a fertilizer in field or green construction in many countries (Zubris and Richards 2005; Talvitie et al. 2017b). On the other hand, wastewaters substantially differ from many natural waters, because they contain large amounts of organic materials, especially cellulosic fibres, which can be easily mixed with some plastic fibres (Lares et al. 2018). As a result, the recovery and examination of MPs become more difficult and time-consuming.

In this context, the objective of the current study was to assess and compare the suitability of frequently used methods for MPs extraction and identification with wastewater and sludge samples collected from a municipal WWTP. As the density (0.9 and 1.6 g/cm^3^) and sensitivity to acidic treatments and high temperatures differ between polymers (Hidalgo-Ruz et al. 2012; Lusher et al. 2017), polymers with different properties were included in the current study. For example, PA and PET are one of the most sensitive polymers to aggressive or acidic treatments with e.g. hydrogen peroxide or sulphuric acid (Lusher et al. 2017). On the other hand, most commonly used polymers, including PP, PE, PVC, PET, PS and PA (PlasticsEurope 2016), are supposed to end up in the environment more often than other polymers. Hence, all these polymers were included in the current study.

In the first part, the recovery of spiked microplastics was investigated with influent and sludge samples with six methods, including filtration (Talvitie et al. 2015), wet peroxide oxidation (WPO) (Lares et al. 2018), degradation with potassium hydroxide (KOH) (Karami et al. 2017), oil extraction procedure (OEP) (Crichton et al. 2017), density separation (Zhang et al. 2016) and a drying-based method (Murphy et al. 2016; only with sludge samples). Seven different MPs were used in spiking. In the second part, two to three methods with highest recovery rates, one with chemical treatment and one or two chemical-free ones, were further adjusted for both wastewater and sludge with the aim of offering effective, environmentally friendly and inexpensive methods, which could serve other researchers involved in MP studies in WWTPs. In addition, staining with Rose Bengal was combined with most effective methods and the utility of staining was assessed. Fourier-transform infrared (FTIR) microscopy and micro-Raman spectroscopy were used in order to assess the possible effects of the selected method on the spiked MPs. To the best of our knowledge, this is the first study to compare suitability of different treatment methods for wastewater and sludge samples collected from WWTP and including both particles and fibres on the recovery rate testing.

## Methods

### Spiked microplastics

**Fig. 1:**
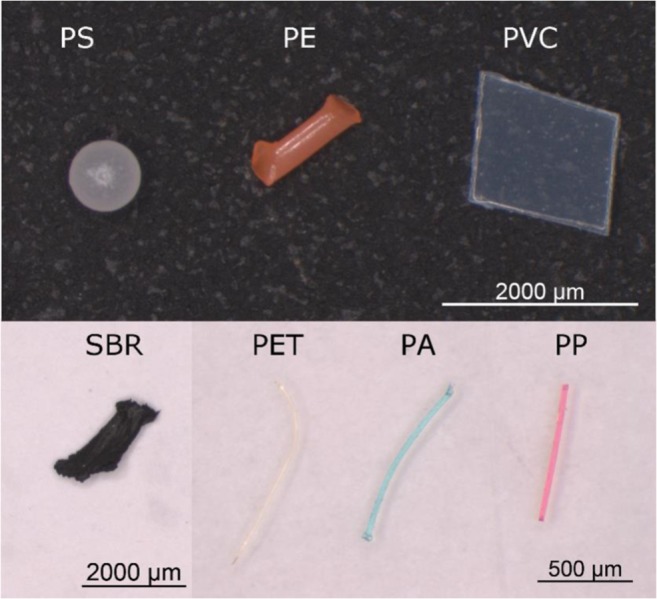
Representatives of spiked microplastics: PS beads; PE, PVC and SBR fragments; PET, PA and PP fibres

Efficiencies of different methods were studied with seven different MPs, including both microplastic particles (MPPs) and fibres (MPFs) (Fig. 1). Altogether, seven different polymers were used in this study, namely (unexpanded) PS, PE, PVC, PET, PA, PP and styrene-butadiene rubber (SBR). Densities of selected MPs varied between 0.9–1.6 g/cm^3^ (Hidalgo-Ruz et al. 2012). The size of each type of spiked MPs (particles and fibres) varied in the ranges listed in Table 1. PS beads were purchased from Sigma-Aldrich and SBR fragments were offered by Saltex Oy. Non-commercial MPs were cut from plastic items and stored in distilled water before spiking. Furthermore, similarly looking MPs (i.e. compared to the spiked ones in this study) were not detected in the WWTP in question during our previous sampling campaign (Lares et al. 2018).

**Table 1:** Details of the microplastics used in the current study

Type	Polymer	Shape	Source	Colour	Longest dimension (μm)	Diameter of fibres (μm)
Particle	PS	Sphere	Commercial	White	650–950	–
	PE	Fragment	Rope	Orange	400–2900	–
	PVC	Flat	Folder	Transparent	300–3100	–
	SBR	Uneven fragment	Recycled rubber	Black	900–3400	–
Fibre	PET	Curved fibre	Fleece textile	Yellow	600–6700	20
	PA	Straight fibre	Yarn	Green	650–3600	30
	PP	Straight fibre	Rope	Red	650–3300	30

### Sample matrix

In order to test different treatment methods with real wastewater and sludge, samples were collected from Kenkäveronniemi WWTP (Mikkeli, Finland) and used as test media. Influent water was collected after 6 mm screen, in the beginning of the grit separation basin, with a 10-L stainless steel bucket attached to a metal wire and poured into 5-L PE containers. Digested sludge (250 mL) was collected directly after digestion into a glass flask. Sampling locations are marked in Figure S2. Samples were stored in dark at + 4 °C until further treatments for maximum of 2 days. According to previous tests conducted in the same WWTP, digested sludge had water content of 97.0% (Lares et al. 2018).

### Sample processing

Sample preparation and examination were done similarly for all of the samples. Subsamples of thoroughly mixed influent and digested sludge were spiked with ten pieces of each one of the seven types, i.e. total of 70 MPs. Model MPs were collected on a Petri dish in a small amount of distilled water, and spiked in 1 L (part I) or 0.1 (part II) of influent or 3 g (wet weight) of digested sludge with small amount of distilled water. Petri dishes were checked under a digital optical microscope (Zeiss, SteREO discover. V8 with Axiocam 503 color) to ensure that all MPs were transferred to the samples. In part I, spiked samples were further treated with six different methods: filtration, WPO, degradation with KOH, OEP, density separation and drying. Three replicates were conducted for each method. All vessels were rinsed three times with distilled water when transferring the samples. Petri dishes and filters were examined for contamination under the microscope before use.

After the treatments, samples were examined twice on nylon/cellulosic/glass fibre filters under the microscope with × 20 magnification and spiked MPs were collected on another petri dish, unless otherwise specified. Only particles and fibres with similar colour and appearance with spiked MPs were counted. For each tested method, the average recovery rates and related standard errors (s.e.m.) were calculated for each MP type, MPPs, MPFs, and all spiked MPs based on the number of recovered MPs.

#### Part II: method adjustment

After several pre-tests including all of the six methods, described in the coming “Compared methods” section, were compared, three methods (one using chemicals and two chemical-free methods) were selected for further optimization, based on the recovery rates calculated in part I (Table 2). Thus, for wastewater, filtration and WPO were selected, with total recovery rates of 83.3% (± 2.5) and 85.2% (± 2.1), respectively. For sludge, drying (85.3 ± 3.4%), filtration (72.4 ± 2.4%) and WPO (90.0 ± 0.8%) were selected.

**Table 2:** Recovery rates (%) of spiked microplastic particles (MPPs) and fibres (MPFs) with investigated extraction methods for spiked wastewater and sludge samples in parts I and II. The suggested methods for wastewater and sludge are set to italics

Method	Sample type	Adjustment	Part	Mesh size (μm)	Recovery of MPPs (%)					Recovery of MPFs (%)				Total recovery of MPs ± s.e.m. (%)
					PS	PE	PVC	SBR	Total **±** s.e.m.	PET	PA	PP	Total **±** s.e.m.	
*Filtration*	Wastewater	Drying at RT	I	250	100.0	96.7	96.7	96.7	97.5 ± 1.4	26.7	86.7	80.0	64.4 ± 4.8	83.3 ± 2.5
		*Drying at RT*	*II*	*25*	*100.0*	*96.7*	*93.3*	*100.0*	*97.5 ± 0.0*	*63.3*	*90.0*	*96.7*	*83.3 ± 3.8*	*91.4 ± 1.7*
		Drying at 45 °C	II	250	100.0	100.0	100.0	100.0	100.0 ± 0.0	23.3	96.7	86.7	68.9 ± 2.9	86.7 ± 1.3
		Drying at 45 °C	II	25	100.0	100.0	100.0	100.0	100.0 ± 0.0	33.3	100.0	96.7	76.7 ± 1.9	90.0 ± 0.8
		Drying at RT + RB	II	25	96.7	96.7	100.0	100.0	98.3 ± 0.8	50.0	96.7	100.0	82.2 ± 6.8	91.4 ± 3.3
	Sludge	Drying at RT	I	250	100.0	90.0	96.7	96.7	95.8 ± 0.8	16.7	63.3	43.3	41.1 ± 6.2	72.4 ± 2.4
		*Drying at RT*	*II*	*25*	*100.0*	*100.0*	*100.0*	*100.0*	*100.0 ± 0.0*	*90.0*	*80.0*	*80.0*	*83.3 ± 3.3*	*92.9 ± 1.4*
		Drying at RT + RB	II	25	100.0	96.7	96.7	100.0	98.3 ± 1.7	66.7	90.0	83.3	80.0 ± 6.7	90.5 ± 3.8
WPO	Wastewater	WPO 75 °C	I	250	96.7	100.0	96.7	100.0	98.3 ± 0.8	30.0	93.3	80.0	67.8 ± 4.0	85.2 ± 2.1
		WPO 50 °C	II	250	100.0	96.7	96.7	100.0	98.3 ± 0.8	26.7	90.0	96.7	71.1 ± 4.4	86.7 ± 2.4
		WPO 50 °C	II	20	100.0	96.7	96.7	100.0	98.3 ± 0.8	30.0	90.0	100.0	73.3 ± 3.8	87.6 ± 2.1
		WPO 50 °C + RB	II	20	100.0	100.0	100.0	100.0	100.0 ± 0.0	56.7	100.0	76.7	77.8 ± 1.1	90.5 ± 0.5
	Sludge	WPO 75 °C	I	250	100.0	96.7	96.7	100.0	98.3 ± 0.8	60.0	86.7	90.0	78.9 ± 1.1	90.0 ± 0.8
		WPO 50 °C^c^	II	250	100.0	96.7	100.0	100.0	99.2 ± 0.8	50.0	83.3	93.3	75.9 ± 3.8	89.1 ± 2.0
		WPO 50 °C^c^	II	20	100.0	96.7	100.0	100.0	99.2 ± 0.8	56.7	86.7	96.7	80.3 ± 5.0	91.0 ± 2.4
		WPO 50 °C + RB	II	20	100.0	96.7	100.0	100.0	99.2 ± 0.8	50.0	86.7	90.0	75.6 ± 8.9	89.0 ± 4.1
KOH	Wastewater		I	250	96.7	100.0	96.7	96.7	97.5 ± 1.4	6.7	100.0	86.7	64.4 ± 2.2	83.3 ± 0.5
	Sludge		I	0	100.0	96.7	96.7	100.0	98.3 ± 0.8	6.7	86.7	83.3	58.9 ± 2.9	81.4 ± 0.8
OEP	Wastewater	a	I	250	100.0	95.0	100.0	100.0	98.8 ± 1.3	25.0	85.0	80.0	63.3 ± 2.7	83.6 ± 2.1
	Sludge		I	0	83.3	96.7	96.7	76.7	88.3 ± 5.5	63.3	93.3	73.3	76.7 ± 3.3	83.3 ± 4.2
Density separation	Wastewater	b	I	250	86.7	43.3	50.0	46.7	56.7 ± 6.7	6.7	36.7	48.9	30.3 ± 3.2	45.4 ± 3.2
	Sludge		I	250	70.0	66.7	76.7	86.7	75.0 ± 1.4	6.7	16.7	26.7	16.7 ± 0.0	50.0 ± 0.8
Drying	Sludge	Without sieving^c^	I	0	100.0	100.0	100.0	100.0	100.0 ± 0.0	16.7	93.3	86.7	65.8 ± 7.9	85.3 ± 3.4
		With sieving^c^	II	20	96.7	93.3	100.0	100.0	97.5 ± 1.4	50.0	73.3	80.0	67.8 ± 6.8	84.8 ± 3.7

*RB* staining with Rose Bengal. Number of replicates = 3

^a^Only two replicates were done

^b^One replicate had nine PA fibres

^c^One replicate had 11 fibres of PA or PP

Adjustments were done based on the size limitations and used temperatures. As the diameter of spiked fibres varied between 20–30 μm, they were able to pass the 250-μm mesh lengthwise. This could explain the low recovery of MPFs (Table 2). Because lower size limitations used in MPs studies has been recommended (Talvitie et al. 2017a; Lares et al. 2018), the lower size limit of sample treatment was decreased from 250 to 20–25 μm in filtration and WPO. Samples sieved with smaller mesh sizes were also examined with higher magnifications (× 31.5). With the introduction of finer mesh sizes, the volume of sieved influent samples was decreased in order to prevent clogging. First, 0.9 L of influent was sieved with the coarse mesh (250 μm), after which 0.1 L of spiked influent was sieved with the whole cascade of filters.

In addition, because yellow and flexible PET fibres were difficult to separate from high loads of cellulose fibres in WWTP samples (and they repeatedly had lowest recovery rates of all spiked MPs), the suitability of staining was studied with adjusted filtration and WPO. Rose Bengal was selected, because it is not toxic and it stains other materials than plastics. Through this way also, the colour of the MPs could be assessed, which is not possible with e.g. Nile Red, because it stains specifically MPs (Fischer et al. 2016). The resistance of model MPs to Rose Bengal was confirmed by staining ten pieces of each of the model polymers with 0.2 mg/mL Rose Bengal solution (4,5,6,7-tetrachloro-2’,4’,5’,7’-tetraiodofluorescein disodium salt, ≥ 80% dye content) according to the method by Ziajahromi et al. (2017).

Results of adjusted methods were compared to the original recovery rates calculated in part I, and the methods with highest recoveries were selected as best methods.

#### Identification

Composition of each spiked MP type was confirmed before optimized treatments with FTIR microscope and micro-Raman spectroscopy. Possible effects of adjusted treatments on identification were also tested by analysing MPs after optimized treatments and staining.

Samples were analysed with FTIR microscope (Spotlight 200i FT-IR microscope system equipped with Spectrum Two, Perkin Elmer) in the reflectance mode using 24 scans and spectral resolution of 4 cm^−1^. Spectra were produced with wavenumbers 600–4000 cm^−1^. Baseline correction, data tune-up and normalization were done for FTIR spectra. Processed spectra were compared to the spectra libraries supplied by Perkin Elmer.

For Raman analyses, micro-Raman spectroscopy (Horiba Jobin Yvon, Labram HR) was used with green laser (514.53 nm). Spectra were produced with wavelengths 300–3000 cm^−1^ using LabSpec 5 software and they were compared to spectra library collected from the literature (Frère et al. 2016; Zhang et al. 2016; Crawford and Quinn 2017).

### Compared methods

Schematic diagram of the setup and detailed descriptions for each method are available in Supplementary Information.

#### Filtration

In part I, a filtration device introduced by Talvitie et al. (2015) was built and used with nylon net with mesh size of 250 μm. Spiked sludge samples were diluted with 3 L of tap water before filtration (Talvitie et al. 2017b), all spiked samples were filtered through the device and filters were carefully transferred into petri dishes. Filters were let dry at room temperature (RT) with a loose foil cover before examination.

In part II, two additional nylon meshes with mesh sizes of 25 and 100 μm were added in the filtration device according to Talvitie et al. (2015). In addition, one set of replicates was dried in oven at 45 °C overnight (15 hours) and other replicates at room temperature (45 hours) in order to assess the differences between different drying procedures. Lid of the petri dishes were kept slightly open in both cases.

Another set of replicates were stained with Rose Bengal. Nylon filters were placed one by one on a glass fibre filter (VWR, Grade 696, porosity 1.5 μm) in a vacuum filtration setup and treated with Rose Bengal solution according to Ziajahromi et al. (2017). Nylon and glass fibre filter were transferred together to a petri dish.

#### WPO

In part I, WPO was conducted according to Lares et al. (2018). Spiked samples were sieved through a metallic test sieve with a mesh size of 250 μm, retained material was rinsed into a beaker and samples were dried in oven at 75 °C. For dried influent samples (0.26–0.28 g dw), 20 mL of 0.05 M FeSO_4_ solution and 40 mL of 30% hydrogen peroxide (H_2_O_2_) were added. After 20-min heating at 75 °C, 20 mL of H_2_O_2_ was added, after which samples were heated for another 30 min. For dried sludge samples (0.15–0.23 g dw), only 20 mL of H_2_O_2_ was added and samples were heated for 30 min. All samples were vacuum-filtrated on gridded membrane filters (Sartorius, cellulose nitrate filter, porosity 0.8 μm) with glass fibre filters (VWR, Grade 696, porosity 1.5 μm) at the bottom for mechanical support and dried in petri dishes with loose foil cover at RT.

In part II, the effect of lower temperature and decreased lower size limit on the recovery was studied with WPO. Lower temperature was studied in order to prevent possible melting of MPs. Spiked influent and sludge samples were sieved through a cascade of test sieves, with mesh sizes of 250 and 20 μm. Retained material was rinsed with small amount of distilled water into glass beakers and dried in oven at 50 °C with a pierced foil cover until dry (approx. 53 h). Samples were further treated as described for part I, except heating at 50 °C during WPO.

For the assessment of staining, 5 mL of Rose Bengal solution was added on the filtered, WPO treated samples and let to react for 5 min. Afterwards, each sample was dried with a vacuum filtration and filter was transferred to a petri dish.

#### KOH degradation

Degradation with 10% KOH solution was conducted according to Karami et al. (2017) with small adjustments. Spiked influent samples were sieved through a metallic test sieve with a mesh size of 250 μm and retained fraction was rinsed into a laboratory glass bottle with a small amount of distilled water. Spiked sludge samples were poured into the bottles without sieving. KOH solution was added into the samples in a proportion of 1:10 (*w*/*v*), samples were maintained in oven at 40 °C for 48 h and vacuum-filtrated on glass fibre filters (VWR, Grade 696, porosity 1.5 μm).

#### OEP

OEP was conducted based on the method by Crichton et al. (2017). They also introduced a method for samples with high organic content, but only the OEP part was tested in this study in order to avoid including many steps, thus preventing possible loss of MPs (Mintenig et al. 2017). The procedure tested previously with seawater was used for spiked and sieved (> 250 μm) influent samples and method introduced for sediment samples was used for spiked sludge samples as Crichton et al. (2017) described.

#### Density separation

Density separation was conducted according to Zhang et al. (2016) using potassium formate (KHCO_2_) solution with density of 1.5 g/cm^3^ (1000 g/L). Spiked influent and sludge samples were sieved through test sieves with 1 mm and 250 μm mesh sizes and MPs were collected with tweezers from the 1-mm sieve. Retained fraction from the 250-μm mesh was rinsed into a glass beaker and dried at 60 °C, after which 150 mL of potassium formate solution was added to each sample and samples were let settle overnight. Supernatants of the samples were vacuum-filtrated on glass fibre filters (VWR, Grade 696, porosity 1.5 μm) and oven-dried for 30 min at 60 °C.

#### Drying

A method based on drying was performed only for sludge samples according to Murphy et al. (2016). In part I, spiked sludge samples were poured into petri dishes and oven-dried at 45 °C for 19 h. In contrast to other samples, these samples were examined three times under the digital optical microscope and small amount of distilled water was added to break down the sludge material during examination.

In part II, the effect of sieving was studied in order to decrease the amount of organic material in sludge samples. Spiked sludge samples were sieved through a test sieve with mesh size of 20 μm. Retained material was rinsed into two petri dishes with small amount of distilled water, because the volume increased during rinsing of the sieve. Samples were further dried and examined similarly as in part I.

### Statistical analysis

Statistical analyses were performed with SPSS (IBM SPSS Statistics Version 25) using non-parametric tests. Kruskal-Wallis test with pairwise comparison was used to assess significant differences between treatment methods by comparing recoveries of MPPs, MPFs and MPs. If distributions of compared methods were different, Welch’s test with Games-Howell post hoc test was performed instead of Kruskal-Wallis. The significance level of all statistical analyses was set at 0.05.

## Results and discussion

According to results from the first part, WPO, drying and filtration were further assessed in the second part. Overall, MPFs had smaller recoveries than MPPs, which was likely caused by their shape (Lares et al. 2018). In the second part, adjusted filtration with three meshes (25, 100 and 250 μm) had highest recovery (83.3%) of MPs for both wastewater and sludge, and it is suggested to be used in further studies of MPs in WWTP-related samples.

### Part I: comparison of different treatment methods

**Fig. 2:**
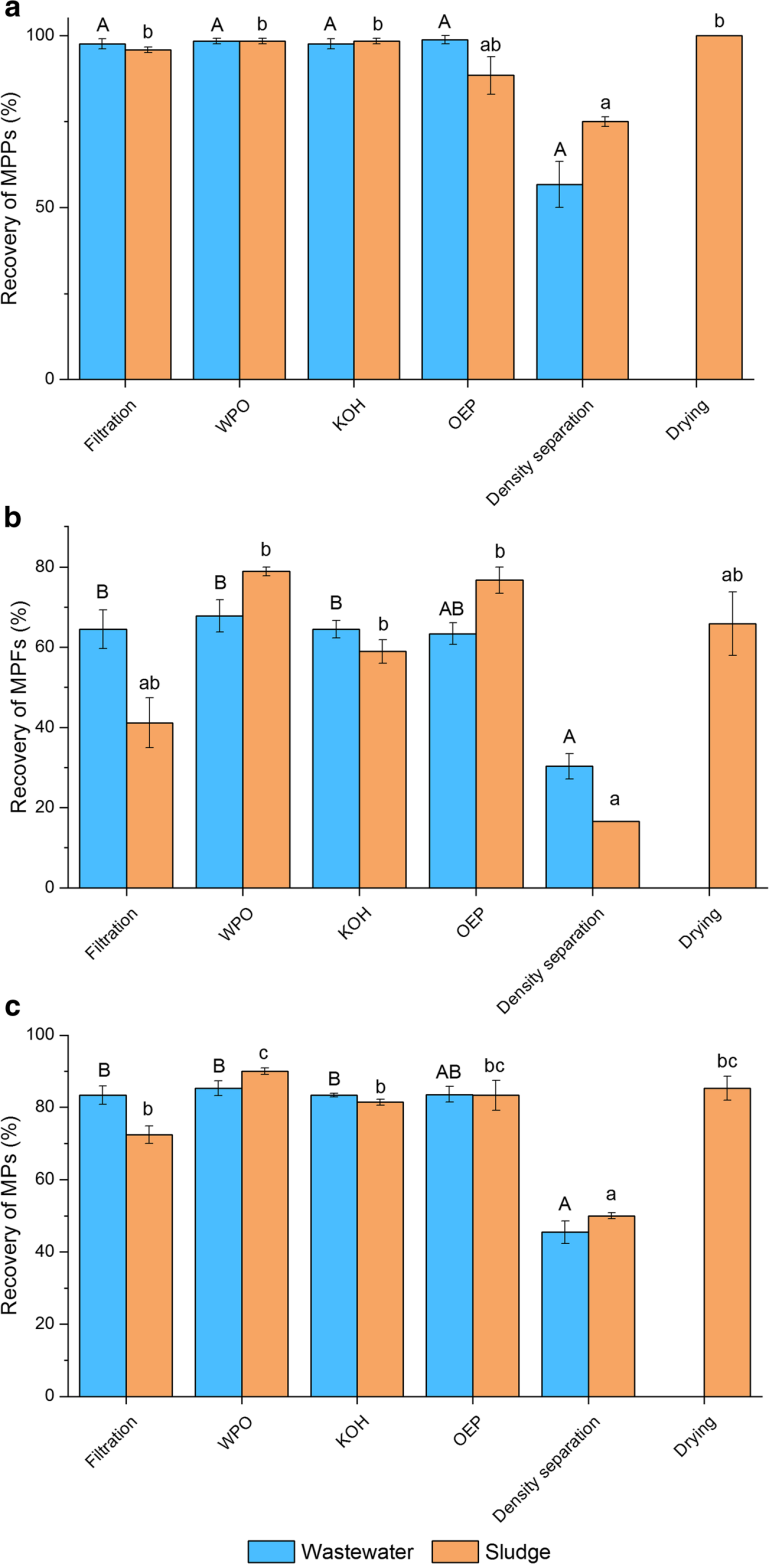
Recovery rates (± s.e.m.) of (**a**) MPPs, (**b**) MPFs and (**c**) total MPs in part I. Blue bars represent wastewater and orange bars sludge samples. Different letters over the bars indicate significant differences among methods. Results were compared only with results gained with same media (wastewater or sludge)

Highest total recovery rates were calculated for WPO (90.0% for sludge and 85.2% for wastewater), drying (85.3% for sludge), OEP (83.6% for wastewater and 83.3% for sludge), filtration (83.3% for wastewater) and KOH treatment (83.3% for wastewater) (Table 2, Fig. 2).

Furthermore, filtration, WPO, KOH treatment, OEP (with wastewater) and drying were efficient methods with MPPs over 250 μm. However, MPFs were more difficult to collect from environmental samples by sieving due to their shape (Lares et al. 2018). Therefore, it was not surprising that the average recovery rates for MPFs varied between 16.7% for density separation (sludge) and 78.9% for WPO (sludge). As the diameter of spiked MPs in the current study varied between 20 and 30 μm, they were able to pass the 250-μm mesh lengthwise.

In addition, advantages and disadvantages of each tested methods were observed (Table 3) and certain methods were justifiably excluded from the second part of the study. OEP was excluded, because oil was not completely removed with detergent and reagent alcohol, which could affect the identification with Raman and FTIR (Crichton et al. 2017). In addition, the size of the outlet of separatory funnel limits the upper size of extracted MPs and larger particles could easily clog the funnel.

**Table 3:** Estimated active and total time consumption (h/sample) together with advantages and disadvantages for different treatment methods with wastewater (W) and sludge (S) samples

Treatment method	Active time (h/sample)	Total time (h/sample)	Advantages (+)	Disadvantages (−)
Filtration	W: 2.0 S: 1.0	W: 50.0 S: 49.0	+ Possible to treat samples immediately in the field + Simple, chemical-free procedure + No visible deformations + Size limits easy to change	− Cellulose fibres not removed − Transferring the filters possible point of losing MPs − Small MPs might pass the sieve while drying; difficult to examine under the filter − Possible contamination from mesh (PA) and device (PVC)
WPO	W: 2.5 S: 2.0	W: 43.0 S: 46.0		− Cellulose fibres only slightly degraded − Toxic chemicals and aggressive reaction − Heat can alter the appearance of MPs − MPs may attach glass surfaces
KOH	W: 2.0 S: 1.5	W: 50.0 S: 50.0	+ Simple procedure + No visible deformations	− Cellulose fibres and other organic material not removed − Organic material attached to MPs; may affect identification − High chemical consumption (alkaline) − Filters clogged quickly
Oil extraction	W: 4.5 S: 3.5	W: 4.5 S: 46.5	+ Canola oil cheap and harmless + No visible deformations	− Cellulose fibres not separated − Multistep procedure − Not suitable for larger MPs − Some oil present after rinsing with alcohol; might affect identification − MPs stick easily on the glass surfaces − Reagent alcohol (methanol) toxic − High static electricity of MPs (especially PE and PS) during examination possibly due to reagent alcohol
Density separation	W: 2.0 S: 1.0	W: 57.0 S: 56.5	+ Simple procedure without harmful chemicals + No visible deformations	− Cellulose fibres (density 1.5 g/cm^3^) not separated − Organic material attached to MPs; may affect identification − 1 mm sieving possible point of losing MPs; dark coloured MPs difficult to separate from organic material with the naked eye − MPs may attach glass surfaces during drying
Drying	S: 3.0	S: 22.0	+ Simple, chemical-free procedure + No visible deformations, but some MPs had brownish colour	− Examination time-consuming and highly based on visual properties − MPs may attach on the walls of the petri dish during drying

Also, density separation was excluded due to the low recovery rate and the fact that cellulose fibres (with density of 1.5 g/cm^3^) were concentrated in the supernatant with MPs. Solid material attached the glass surfaces during drying, which could have affected on the recovery of MPs. Because cellulose could not been separated based on its density, this method was excluded from further optimization. We agree that density separation could be more useful with sediment samples, where it could separate denser mineral particles from MPs (Zhang et al. 2016).

KOH treatment of spiked samples gave slightly lower total recoveries than WPO for both test media (significant difference only with sludge, *p* = 0.011). Since our objective was to optimize only one method using chemicals, KOH treatment was excluded from further studies at this point. In addition, lots of organic material were left in samples after the KOH treatment.

Some problems were noticed with all tested treatment methods. First, none of the tested procedures were able to remove all cellulose fibres from samples. Overall, cellulose is an organic polymer, which is relatively stable against most of the mild chemical treatments. If harsh chemicals would be used to degrade cellulose, also MPs might be prone to chemical degradation. Therefore, staining was also tested in the second part of the study in order to separate non-plastic particles and fibres from microplastics. Another option could have been enzymatic degradation of cellulose, which was not found to be useful with influent samples (Lares et al. 2018), and would thus need further optimization before its utilization in MPs studies.

Second, PET fibres had the lowest recovery rates during the whole study. Those fibres were more flexible than PA and PP fibres, which may have allowed them to pass sieves more easily. They were also difficult to separate from cellulose fibres during the visual examination. In addition, the light colour of the filters or microscope’s sample holder might cause some errors in the results, when MPs are collected visually. Light-coloured MPs are more easily recognized from dark-coloured background and vice versa. However, selecting the optimal background colour (i.e. colour of the filter) to analyse all MPs would probably not be possible.

When recovery rates of methods for particulate material are discussed, it should be noticed, that recovery rates depend highly on the used mesh sizes and particles in question. If the portion of spiked large particles increases, the recovery rate of the method will also increase. If only fibres are studied, the total recovery rate would likely decrease. This was taken into account in the current study, as recovery rates were also counted separately for particles and fibres. In addition, even though only the number of recovered fibres was counted, it seemed that longer PET fibres were counted more often than shorter ones. As not all of the MPs were measured before spiking, this was not further examined and discussed.

In addition, average time consumptions of different methods are listed in Table 3. Active time consumptions for spiked wastewater and sludge samples varied between 1.0 and 4.5 h. OEP, with its multistep procedure, needed clearly more active time than other treatments. Total time needed for treatment, drying and examination varied between 4.5 and 57.0 h. Nevertheless, total time consumptions were highly dependent on the time used for drying and they were also dependent on the water addition caused by spiking. Drying of any filter at low temperature (e.g. 45 °C) would decrease both the time needed for drying and risks for contamination, as samples would be exposed to aerial contamination for a shorter period. Overall, active time consumptions were more representative in this case.

### Part II: adjusted methods

**Fig. 3:**
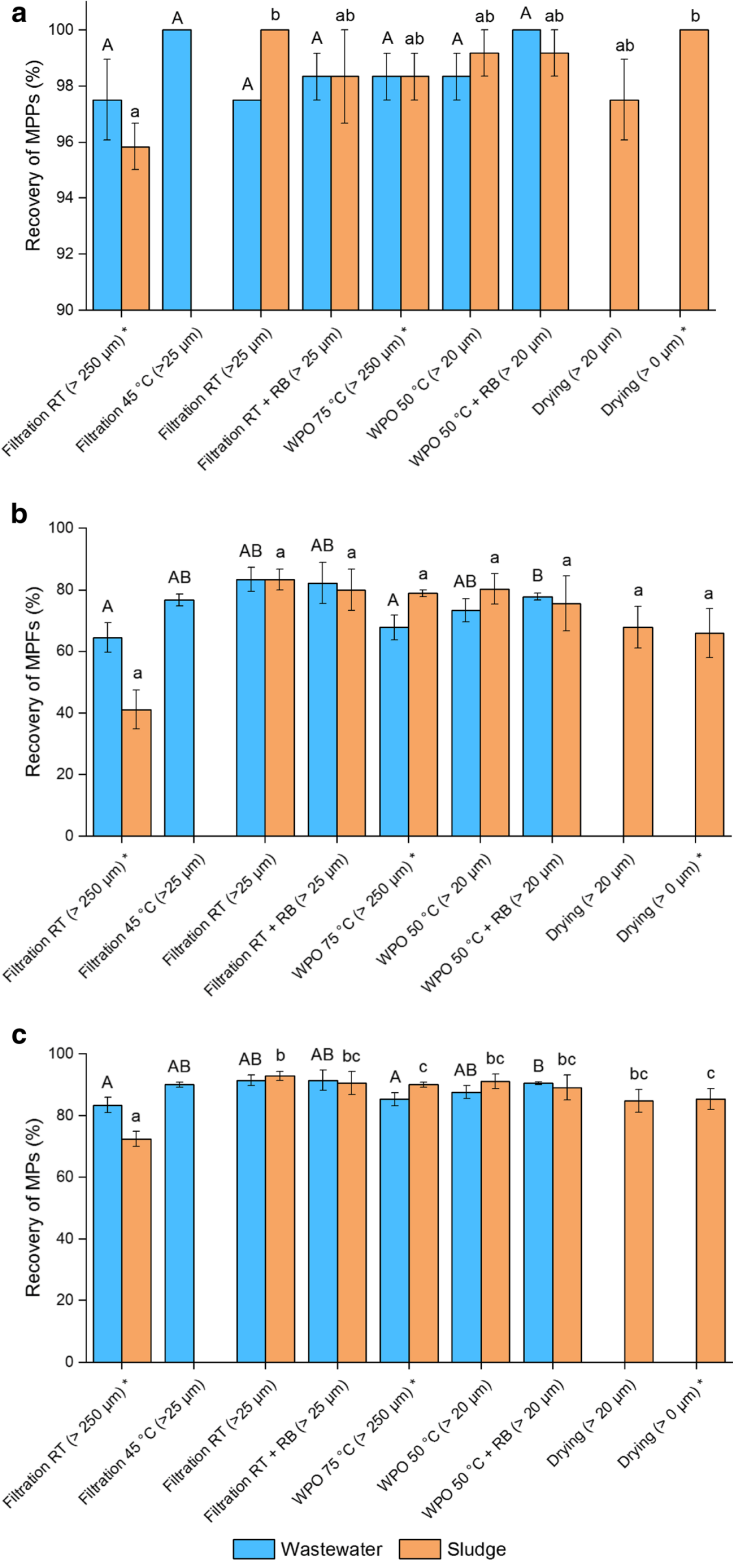
Recovery rates (± s.e.m.) of (**a**) MPPs, (**b**) MPFs and (**c**) total MPs for adjusted methods. Blue bars represent wastewater and orange bars digested sludge samples. Different letters over the bars indicate significant differences among methods. Results were compared only with results gained with same media (wastewater or sludge). *results from part I, RT room temperature, RB staining with Rose Bengal

Total recoveries of adjusted methods varied between 84.8 and 92.9% (Table 2) and filtration was found to be the most efficient method for both wastewater (91.4% ± 1.7) and sludge (92.9% ± 1.4). Filtration had also highest recovery rate for MPFs; 83.3% (± 3.8) for wastewater and 83.3% (± 3.3) for sludge (Fig. 3). Nonetheless, none of the adjusted methods differed significantly from others (all pairwise Kruskal-Wallis comparisons or Games-Howell post hot tests *p* > 0.05). This means that by using either one of these adjusted methods, rather similar types and number of MPs would be counted in case of the size of studied MPs is similar with spiked MPs used in the current study. This could ease the comparison of already reported abundances of different kinds of MPs.

The narrow nature of fibres was taken into account in part II, as the lower size range of treatment methods was decreased to 20–25 μm. Overall, recovery rates were slightly higher with decreased mesh size of cascade sampler, as meshes below 250 μm collected 0–18.6% of spiked MPs, all of which were fibres. This difference was significant only for sludge between filtration at RT, with and without staining (pairwise Kruskal-Wallis *p* = 0.046 and *p* = 0.043, respectively). If size of spiked MPs had been smaller, that proportion of MPFs on smaller-sized meshes would presumably have been larger. On the other hand, when focusing on the time consumptions of different methods, decreasing the lower size limit increased the time needed for treatments and examination due to multiple size fractions per sample. Overall, it should be noticed, that when the lower size limit is increased, the treatment and examination of samples are quicker and easier, but the results for MPs concentrations are less accurate. In addition, handling of smaller particles with tweezers is difficult, and therefore new reliable methods must be developed in order to include wider particle fraction into further studies.

#### Filtration

Drying at 45 °C did not have a significant effect on the MPs recovery, but it decreased the time needed for treatment. If time is limited, we would suggest drying in oven using low air circulation and having lids slightly open. No visible deformations were noticed in spiked MPs, except for one PS bead, which had a brownish colour after the treatment.

Samples were fractionized by size more easily with filtration device, when they contained fewer large-sized materials. This was clear when filtered samples of influent and sludge were compared. More fibres were counted from smaller sized meshes with sludge samples than with influent samples, which contained higher amount of cellulose fibres with the ability to retain other particles and fibres on the 250 μm mesh.

#### WPO

Sieving with a cascade of sieves was slightly more time-consuming and caused double amount of work with WPO procedure itself. Only few fibres were counted from the samples sieved with 20 μm. Even though H_2_O_2_ treatment at 50 °C has been reported to cause colour changes in PET fragments (Karami et al. 2017), no colour changes were noticed in any of the spiked PET fibres in present study. Only deformations were noticed in spiked MPs, along with shinier surfaces and/or brownish colour in some of the PS beads.

#### Drying

Due to the sieving, time needed for drying the samples increased considerably. Each sieved sample was divided into two petri dishes before drying, which increased the possible loss of MPs and risk of contamination (not assessed in this study). Few brownish PS beads were collected also from the dried sludge samples, but no other deformations were noticed. As sieving did not improve the recovery compared with the original method (Murphy et al. 2016), we would not recommend adding a sieving step to this method. Based on our observations, larger volume of the samples caused by rinsing of the sieves together with drying might cause MPs to attach on the vertical surfaces of petri dish, thus further complicating the examination of MPs.

#### Staining

Staining with Rose Bengal was not found to be particularly practical. Under the staining procedure conducted in this study, cellulose fibres were not stained pink like some other organic materials like wool and sludge. Overall, the recovery rates of stained samples did not differ significantly from non-stained samples (Table 2, Fig. 3). In addition, Rose Bengal caused slight modifications in the colour of some PS and PVC fragments, especially when staining was conducted after WPO treatment. This is inconsistent with previous studies, where Rose Bengal was found to be suitable for PS and PE (Ziajahromi et al. 2017). Discolouration noticed in the current study might be due to the effect of H_2_O_2_ on the surface of the MPs.

### Effects of suggested method and staining on identification of microplastics

Neither filtration nor staining caused any changes in the identification of particles or fibres, which was confirmed with μ-Raman and μ-FTIR, respectively. Still, it is essential to consider that some of the spiked MPs were difficult to analyse with methods used in this study. SBR fragments were not possible to identify reliably with neither μ-FTIR nor μ-Raman, probably due to the light-absorbing nature of black rubber (Ribeiro-Claro et al. 2016). μ-FTIR was able to give identifiable spectra for all other model MPs. Nevertheless, it was more suitable for fibres, as identification of thick MPPs with μ-FTIR in transmittance or reflectance mode was challenging. Raman analysis in turn gave identifiable spectra to most of the spiked fragments (PS, PE and PVC) and PET fibres. All spiked fibres (PET, PA and PP) were still somewhat problematic to identify with Raman. Because all of the fibres were colourful, difficulties were probably caused by their fluorescence, which can be due to pigments or dyes used in plastics (Löder and Gerdts 2015; Käppler et al. 2016; Ribeiro-Claro et al. 2016).

Käppler et al. (2016) previously discussed the advantages and disadvantages of FTIR and Raman analysis and suggested using both methods in optimized analysis of unknown particles and fibres. Raman analysis is not dependent on the thickness of the particle, but it is more sensitive for the before-mentioned fluorescence and for the chemical transformation of particle surface. As Song et al. (2015) suggested, identification of a wider set of samples should be based on both microscopic and spectroscopic analysis. First, different particles and fibres should be identified with FTIR or Raman and then the microscopic analysis could be done based on the information gained from the spectroscopic analysis. Nevertheless, there would still be a risk of misidentification, especially with smaller sized particles.

### Application of methods to real samples

#### Suggested method: filtration

As filtration (with mesh sizes 25, 100 and 250 μm) was found to be the best method based on the observations and recovery of both MPPs and MPFs, we would suggest using the filtration device, previously introduced by Talvitie et al. (2015), with both wastewater and sludge samples. Its advantages are low costs and zero use of chemicals, which make this method feasible for laboratories around the world.

When filtration is conducted with filtration device and nylon filters (Talvitie et al. 2015), also following issues need to be taken into account. Meticulous rinsing of sampler and filtration device with MP-free water is essential before and after the sampling. Connector parts should be rinsed carefully with water before transferring the filter, as some material can be attached to the connector’s seal. Sludge samples should be mixed with only a small volume of water (e.g. 1 g ww/100 mL). As a disadvantage of this method, both nylon mesh and filtration device (PVC) are possible sources of contamination, and the condition of the device need to be checked before use. Therefore, any MPPs similar to the used PVC and nylon parts should be excluded from results. If possible, non-plastic materials, like metal or glass, should be preferred in order to diminish possible contamination from equipment.

Pre-testing of selected media is also highly recommended to be able to select a suitable sample volume and to prevent clogging of the filtration device. In addition, control samples should be conducted for assessing both procedural and aerial contamination. A chart of the filtration procedure is depicted in SI (Fig. S10).

#### General notes

For future MPs studies, we recommend to pay attention to some general issues. First, in order to decrease the risk of contamination and to increase the recovery rates of the examination, method testing with spiked samples before collecting real samples is highly recommended. It should also be noticed that reported recovery rates are accurate only for the applied protocols. Consequently, recovery rates should always be studied and reported together with the MP concentrations with respect to the adopted analytic procedure (whether adjusted from previous methods or completely new ones).

Second, rather large MPs were used in these experiments. Even though the procedure would allow collection of very small MPs (< 100 μm), there would be a risk of missing them during the microscopic examination before identification with Raman or FTIR microscope. Therefore, the lower size limitation of counted MPs is partly dependent on the available magnification of the microscope. Nevertheless, there are always smaller micro- and even nanoplastics, which will pass the sieves or filters and become excluded from the reported MP concentrations. We would suggest to draw the line of lower size limitation around 20 to 25 μm, as most of the cloth fibres have diameter above that, and those size limitations has already been used in some previous publications (Talvitie et al. 2015; Zhang et al. 2016; Karami et al. 2017; Leslie et al. 2017; Mintenig et al. 2017; Quinn et al. 2017; Sillanpää and Sainio 2017; Talvitie et al. 2017a; Talvitie et al. 2017b; Ziajahromi et al. 2017; Dris et al. 2018). MPs in that size fraction (20 to 100 μm) would anyway need specific instrumentation for examination and handling.

Third, because MPFs cover majority of MP pollution in municipal WWTPs (Talvitie et al. 2015; Leslie et al. 2017; Sillanpää and Sainio 2017; Ziajahromi et al. 2017; Lares et al. 2018), it is essential to include fibres in MPs studies conducted in both WWTPs and in different parts of exposed ecosystems (water, sediments, soil, etc.).

In addition, this study focused only on assessing the efficiency of different methods used in preparations of wastewater and sludge samples for MPs analysis. Also the sampling methods have significant impact on the counted number of MPs related to the real amount of MPs in the samples. Setälä et al. (2016) and Barrows et al. (2017) compared MP concentrations in seawater samples collected from same areas either with Neuston tow net or as grab samples. Because Neuston nets usually have the mesh size of 330–335 μm, and for grab samples the lower size limitation can be set as small as necessary, MP concentrations were much higher in grab samples than in Neuston net samples. Neuston net is also not suitable for wastewater sampling, where samples are collected from specific and limited areas. Other possible sampling methods for wastewater are pumping, composite samplers and surface filtering assemblies (Talvitie et al. 2015; Carr et al. 2016; Talvitie et al. 2017a; Talvitie et al. 2017b). In WWTPs, the sampling procedure should also consider the various operating conditions in different parts of the treatment process (Lares et al. 2018).

## Conclusions

In the light of the present investigation, we would suggest conducting future wastewater and sludge sample treatments to recover MPs using the previously introduced filtration device. Its advantages are low cost and zero use of chemicals, which make it feasible for all laboratories around the world. Staining methods could be useful in separating MPs from other materials. Nevertheless, according to current study Rose Bengal is not suitable for separating cellulose and plastic fibres.

With any kind of samples, the risk of losing or destroying MPs should be decreased by avoiding harsh chemical treatments, high temperatures or multistep treatment procedures. Recommended filtration method did not have an impact on the identification of the model MPs, and according to our results, it had high recovery rates for both microplastic particles and fibres.

For future research studies on MPs, in order to decrease the risk of contamination and increase the recovery rates of the process, it would be extremely important to run pre-tests with spiked samples before collecting real samples for analyses, in order to assess the accuracy of the applied methodology and to report the final results accordingly.

## **Electronic supplementary material**

Representations and detailed descriptions of tested methods, flow diagram of Kenkäveronniemi WWTP, FTIR and Raman spectra for spiked MPs, diagram of treatment procedure for filtration device.

ESM1 (DOCX 1.87 mb)
