# Supplementary material for: Intercomparison study on commonly used methods to determine microplastics in wastewater and sludge samples
Source: Environ Sci Pollut Res Int. 2019 Mar 2;26(12):12109–22. doi: 10.1007/s11356-019-04584-6 (PMC6476832; doi:10.1007/s11356-019-04584-6)
Supplement: Supplementary file 1 — (DOCX 1.87 mb) [file 11356_2019_4584_MOESM1_ESM.docx]

**Supplementary Information**

**Intercomparison study on commonly used methods to determine microplastics in wastewater and sludge samples**

Mirka Lares, Mohamed Chaker Ncibi, Markus Sillanpää, and Mika Sillanpää

Number of pages: 9

Number of figures: 9

**
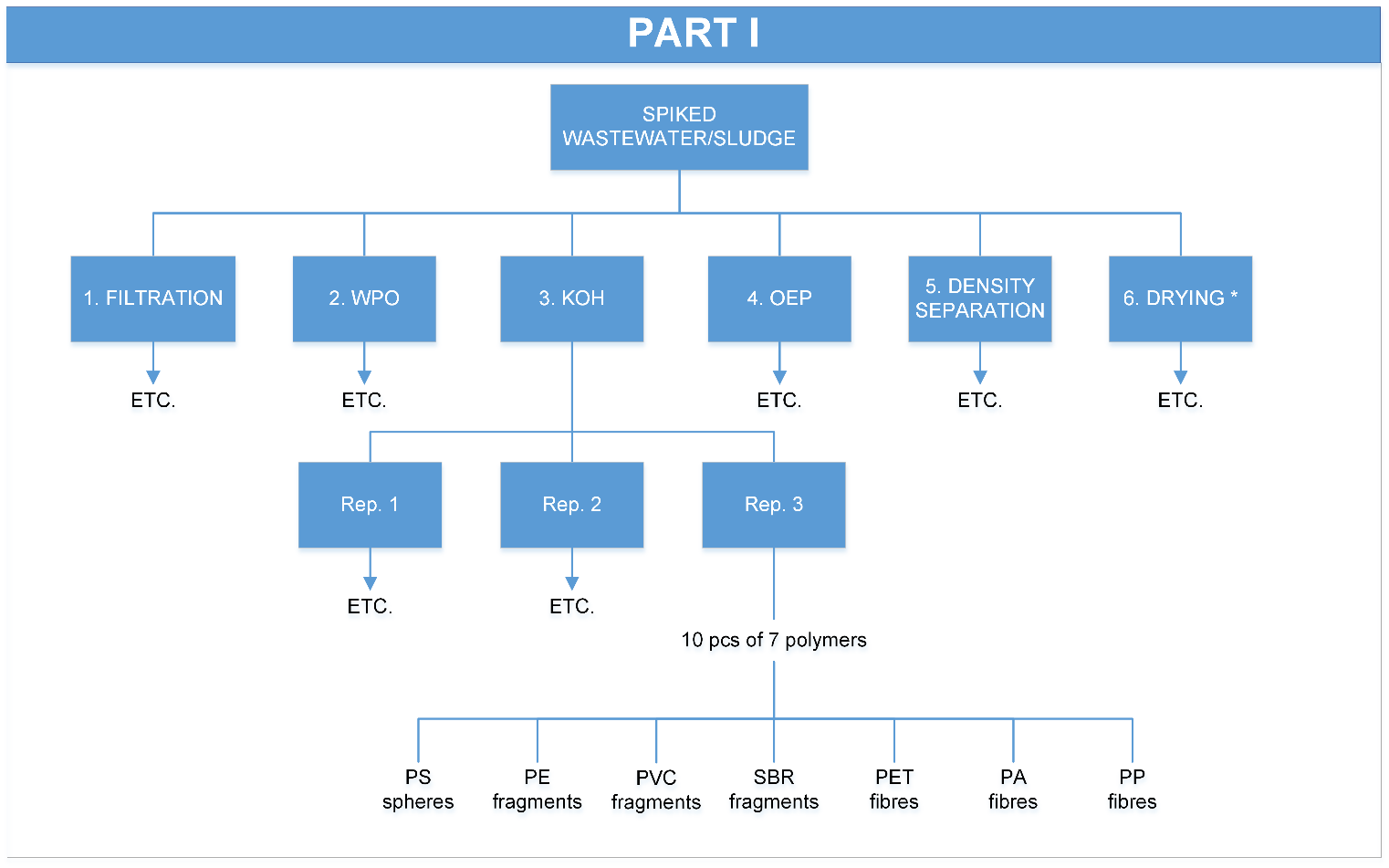
**

**
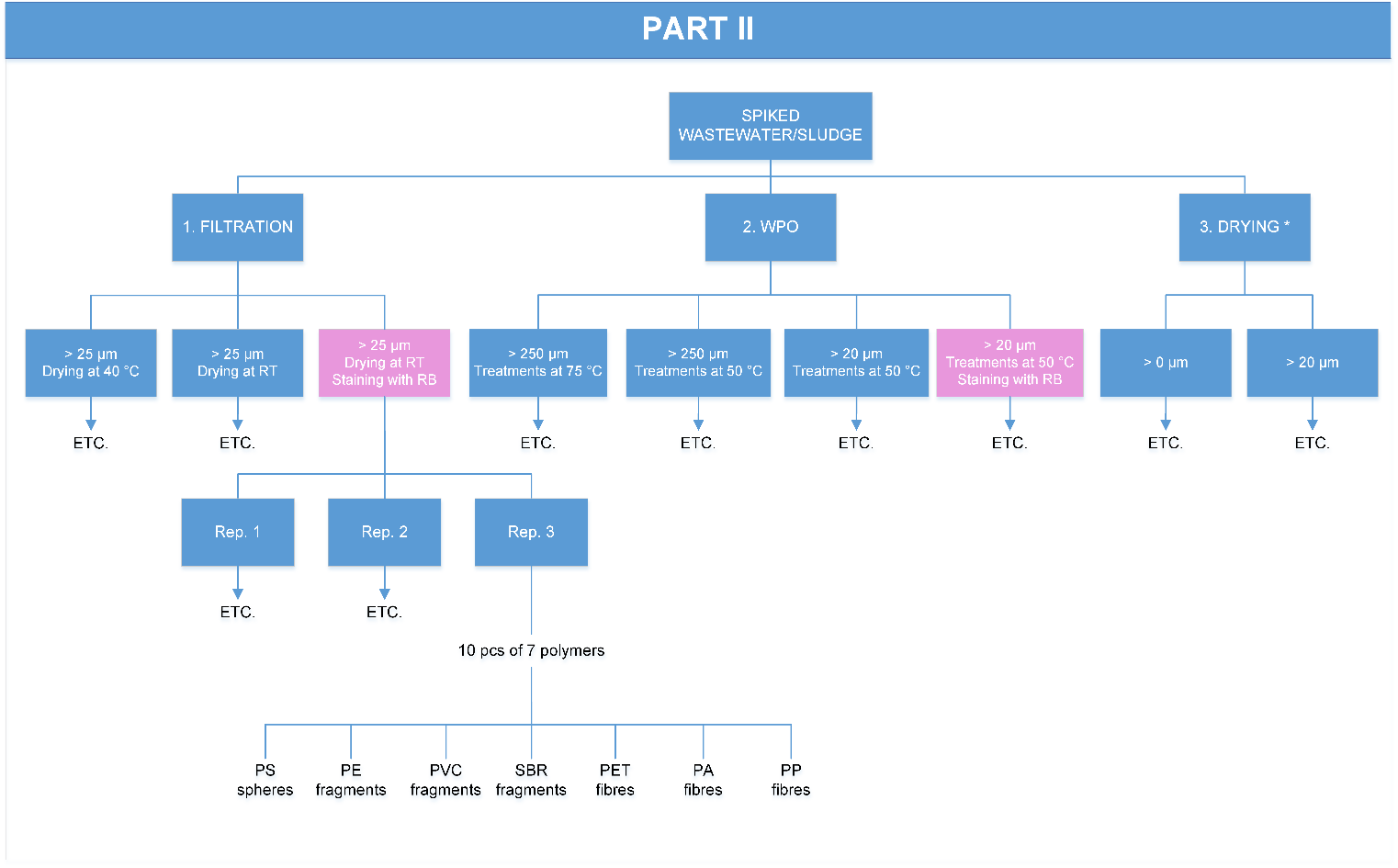
**

**Figure S1** Representation of the conducted experiments in parts I and II. *Drying was only tested with sludge samples


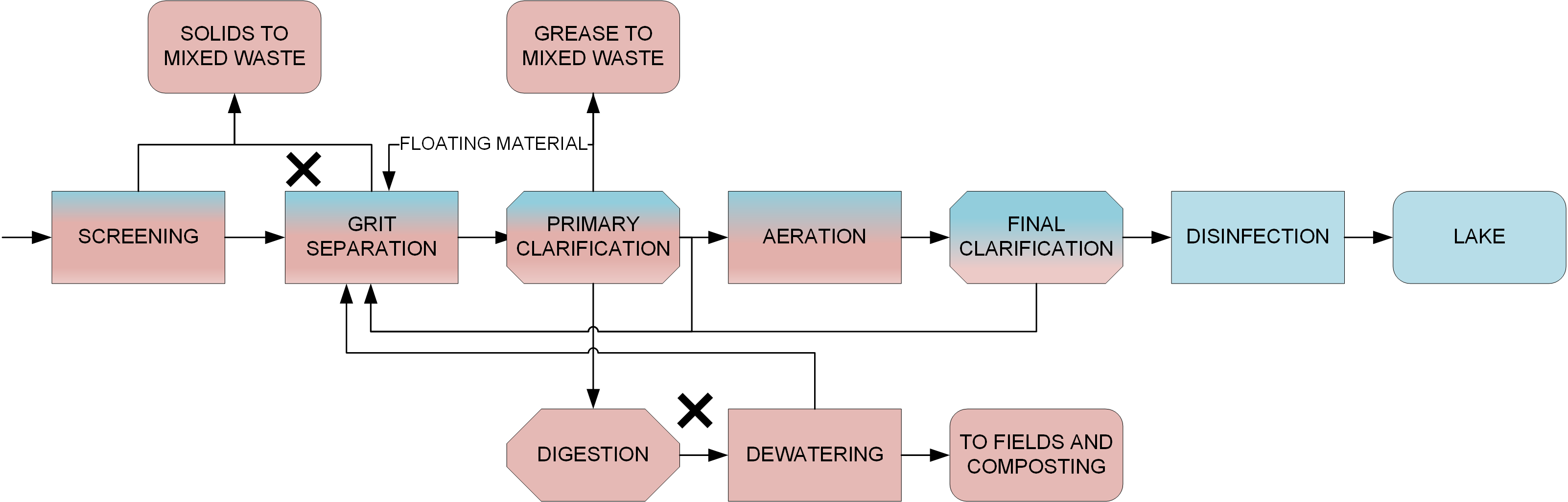


**Figure S2** Flow diagram of the wastewater treatment process in Kenkäveronniemi WWTP, with sampling locations for wastewater and sludge (**Χ**)

**PART I: Detailed descriptions for each method tested with spiked WWTP samples**

**Filtration.** A filtration device, introduced by Talvitie et al. (2015), was built using clear PVC tube (diameter of 7.5 cm) and connectors. Nylon net, with mesh size of 250 µm, was placed between connector parts. Spiked samples were sieved through the filtration device, the walls of the tube were rinsed with tap water and the filter was carefully transferred into a petri dish. Spiked sludge samples were diluted with 3 L of tap water before sieving (Talvitie et al. 2017). Filters were let dry in room temperature with a loose foil cover before examination.

**Wet peroxide oxidation.** Wet peroxide oxidation (WPO) was conducted according to Lares et al. (2018). Spiked samples of influent and digested sludge samples were sieved through a test sieve, with mesh size of 250 µm, and remaining material was rinsed into a glass beaker. Samples were dried in oven at 75 °C with pierced foil cover for approximately 20 h, i.e. until dry.

For influent samples, 20 mL of 0.05 M FeSO_4_ solution and 40 mL of 30 % hydrogen peroxide (H_2_O_2_) was added into the beakers and mixture was left to settle for 5 minutes in room temperature. After 20 min of heating at 75 °C, another 20 mL of H_2_O_2_ was added and samples were heated for additional 30 minutes. Small amount of distilled water was added to slow down the reaction, if reaction started to overheat. For dried sludge samples, only 20 mL of H_2_O_2_ was added together with 20 mL of 0.05 M FeSO_4_ solution, and solution was heated for 30 minutes at 75 °C.

After aforementioned treatments samples were cooled down for at least 10 minutes and collected with vacuum filtration on a gridded membrane filters (Sartorius, cellulose nitrate filter, porosity 0.8 µm) with glass fibre filters (VWR, Grade 696, porosity 1.5 µm) at the bottom for mechanical support. Filters were dried in room temperature with loose foil cover.

**KOH degradation.** Degradation with 10% potassium hydroxide (KOH) solution was conducted according to Karami et al. (2017) with small adjustments. Spiked influent samples were sieved through a 250-µm test sieve and remaining fraction was rinsed into a previously weighed laboratory bottle with a small amount of distilled water. Spiked sludge samples were collected in laboratory bottles without sieving and dried in oven at 40 °C for 16 hours to reduce the higher volume caused by spiking. Drying part would not be necessary with real sludge samples. Potassium hydroxide (10%) solution was added into the samples in a proportion of 1:10 (w/v). Samples were maintained in oven at 40 °C for 48 hours, after which they were vacuum filtered on double glass fibre filters (VWR, Grade 696, porosity 1.5 µm). Filters were not dried before examination.

**Oil extraction procedure.** Oil extraction was constructed based on the method by Crichton et al. (2017). Spiked influent samples were sieved through a 250 µm test sieve and remaining material was rinsed into glass beakers. Samples were rinsed with 200 mL of distilled water into a separatory funnel and 10 mL of canola oil was added. Samples were shaken vigorously for 30 seconds and let settle until the oil layer was separated. Water layer was discarded, samples were shaken and let settle similarly for another time, after which water layer was discarded from the funnels. Oil layer was vacuum filtered on a similar set of filters as with WPO treatment. Separatory funnel was rinsed twice with 25 mL of 4 % non-foaming detergent (Deconex 22 LIQ-x) and emptied to the same filter. Filters were incubated twice in 10 mL of reagent alcohol (90 % ethanol, 5 % methanol and 5 % isopropanol) for 10 min. Finally filters were wetted with small amount of distilled water before transferring it into a petri dish. Filters were not dried before examination.

Accordingly, spiked sludge samples were dried in 100 mL Erlenmeyer flasks at 50 °C for 43 hours, i.e. until most of the additional water due spiking was evaporated. Drying could also have been compensated with smaller addition of distilled water in the next step. 50 mL of distilled water was added to dried sludge samples and flasks were shaken vigorously to wet the sludge. 5 mL of canola oil was added, flasks were shaken for 30 s and let settle until oil layer was completely separated. Samples were transferred into separatory funnels, and the rinsing of Erlenmeyer with water and canola oil was repeated. Flasks were rinsed twice with 50 mL of distilled water and twice with 30 mL of distilled water, which were all poured into the separatory funnel. After these steps samples were treated like wastewater samples by mixing the sample and letting it separate before discarding the water layer.

**Density separation.** Density separation was conducted according to Zhang et al. (2016) using potassium formate (KHCO_2_) solution. Spiked influent and sludge samples were sieved through a 1 mm and 250 µm test sieves, after which potential microplastics were collected with tweezers from 1 mm sieve on a petri dish. Remaining fraction from the 250-µm sieve was rinsed into a glass beaker with small amount of distilled water. Samples were dried at 60 °C until dry (approximately 40 hours). 150 mL of potassium formate solution (1000 g/L) with density of 1.5 g/cm^3^ was added to each sample and samples were let settle overnight. Glass rods were used for releasing dried material from the bottom of the beaker. Anyhow, some material was still stuck on the bottom and after the separation time some dried material was still as chunks in the samples. Supernatants were vacuum filtrated on glass fibre filters (VWR, Grade 696, porosity 1.5 µm) and oven-dried in petri dishes with their lids partly open for 30 minutes at 60 °C.

**Drying.** A method based on drying was tested only with sludge and it was performed according to Murphy et al. (2016). Spiked sludge samples were dried in oven at 45 °C for 19 hours with pierced foil cover. Samples were examined three times under digital optical microscope using tweezers and spiked MPs were collected on another petri dish with tweezers. Small amount of distilled water was added to break up the sludge material during examination.





**Figure S3** Raman spectra for PS spheres; untreated, stained with Rose Bengal, filtered with water and filtered with sludge





**Figure S4** Raman spectra for PE fragment; untreated, stained with Rose Bengal, filtered with water and filtered with sludge





**Figure S5** Raman spectra for PVC fragment; untreated, stained with Rose Bengal, filtered with water and filtered with sludge





**Figure S6** Raman spectra for SBR fragment; untreated, stained with Rose Bengal, filtered with water and filtered with sludge

**

**

**Figure S7** FTIR spectra for PET fibre; untreated, stained with Rose Bengal, filtered with water and filtered with sludge

**

**

**Figure S8** FTIR spectra for PA fibre; untreated, stained with Rose Bengal, filtered with water and filtered with sludge

**

**

**Figure S9** FTIR spectra for PP fibre; untreated, stained with Rose Bengal, filtered with water and filtered with sludge


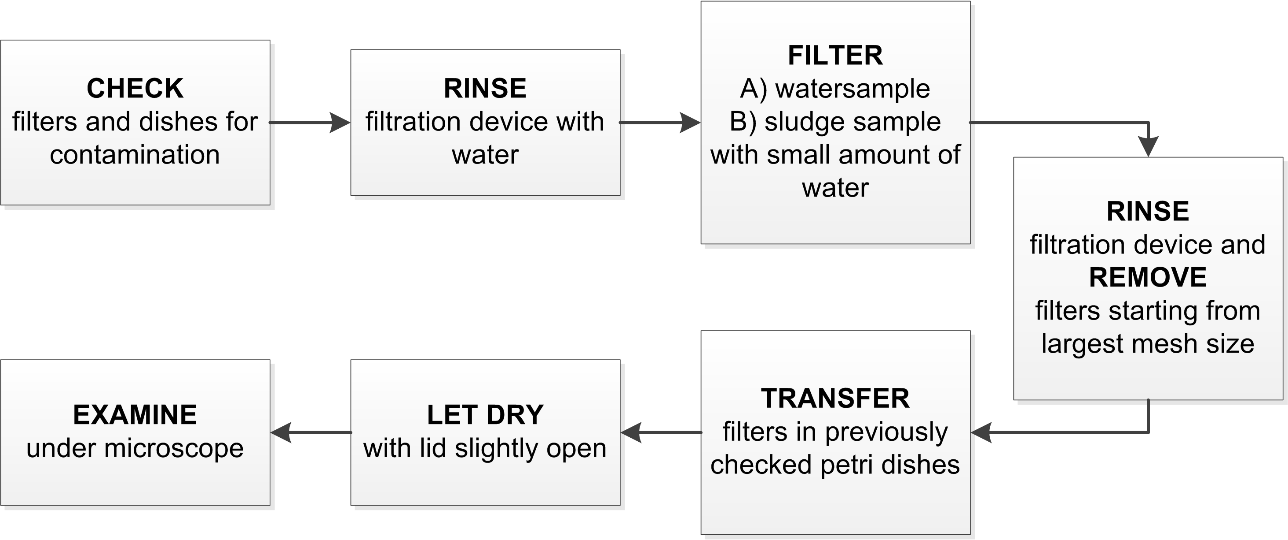


**Figure S10** Treatment procedure using filtration device for wastewater and sludge samples

**REFERENCES**

Crichton EM, Noël M, Gies EA, Ross PS (2017) A novel, density-independent and FTIR-compatible approach for the rapid extraction of microplastics from aquatic sediments. Anal Methods 9(9):1419–1428. https://doi.org/10.1039/c6ay02733d

Karami A, Golieskardi A, Choo CK, Romano N, Ho YB, Salamatinia B (2017) A high-performance protocol for extraction of microplastics in fish. Sci Total Environ 578:485–494. https://doi.org/10.1016/j.scitotenv.2016.10.213

Lares M, Ncibi MC, Sillanpää M, Sillanpää M (2018) Occurrence, identification and removal of microplastic particles and fibers in conventional activated sludge process and advanced MBR technology. Water Res 133, 236–246. https://doi.org/10.1016/j.watres.2018.01.049

Murphy F, Ewins, C, Carbonnier F, Quinn B (2016) Wastewater treatment works (WwTW) as a source of microplastics in the aquatic environment. Environ Sci Technol 50(11):5800–5808. https://doi.org/10.1021/acs.est.5b05416

Talvitie J, Heinonen M, Pääkkönen J-P, Vahtera E, Mikola A, Setälä O, Vahala R (2015) Do wastewater treatment plants act as a potential point source of microplastics? Preliminary study in the coastal Gulf of Finland, Baltic Sea. Water Sci Technol 72(9):1495–1504. https://doi.org/10.2166/wst.2015.360

Talvitie J, Mikola A, Setälä O, Heinonen M, Koistinen A (2017) How well is microlitter purified from wastewater? – A detailed study on the stepwise removal of microlitter in a tertiary level wastewater treatment plant. Water Res 109:164–172. https://doi.org/10.1016/j.watres.2016.11.046

Zhang K, Su J, Xiong X, Wu X, Wu C, Liu J (2016) Microplastic pollution of lakeshore sediments from remote lakes in Tibet plateau, China. Environ Pollut 219:450–455. https://doi.org/10.1016/j.envpol.2016.05.048
